# Global Road Safety Week — May 6–12, 2013

**Published:** 2013-05-03

The United Nations (UN) General Assembly has declared the week of May 6–12, 2013, as Global Road Safety Week. This year the week is dedicated to pedestrian safety. More than 5,000 pedestrians are killed on the world’s roads each week, and pedestrians comprise nearly one quarter of global road deaths annually (1). The vast majority of pedestrian deaths occur in low-income and middle-income countries.

The goal of this year’s observance is to draw attention to the need to provide safe, reliable, and accessible facilities for all pedestrians. The World Health Organization (WHO) is coordinating Global Road Safety Week efforts and recommends increased implementation of strategies known to save pedestrians’ lives, including 1) installing and/or upgrading crosswalks, sidewalks, overpasses, underpasses, raised medians, and road signs and signals; 2) slowing vehicle speeds by “calming” streets with speed bumps and rumble strips; 3) enforcing laws against speeding and distracted driving; 4) creating walking streets or pedestrian zones; 5) improving mass transit route design and access; 6) improving lighting around pedestrian crossings; and 7) enhancing the visibility of pedestrians through the use of reflective materials.

WHO, in collaboration with the CDC and other partners, will release a report in May 2013 regarding “best practices” for pedestrian safety outlining the global problem, risk factors, and interventions to prevent or reduce pedestrian injuries around the globe (2).

Global Road Safety Week is part of the larger UN Decade of Action for Road Safety 2011–2020 activities, aimed at saving 5 million lives on the road by the year 2020. Additional information about Global Road Safety Week, the UN Decade of Action for Road Safety, and ideas on how to get involved in promoting pedestrian safety are available from WHO at http://www.who.int/roadsafety/week/2013/en/index.html. Information on CDC’s efforts to improve global road safety is available at http://www.cdc.gov/features/globalroadsafety, and resources from CDC for preventing road traffic injuries are available at http://www.cdc.gov/motorvehiclesafety and http://www.cdc.gov/winnablebattles/motorvehicleinjury.
